# Presentation, Diagnosis, and Acute Treatment of Secondary Hemophagocytic Lymphohistiocytosis: A Case Report

**DOI:** 10.7759/cureus.98913

**Published:** 2025-12-10

**Authors:** Alexandra Bartholomew, Michael Connick, Catherine Loehr, Shane Sanne

**Affiliations:** 1 Department of Internal Medicine, Louisiana State University Health Sciences Center, New Orleans, USA

**Keywords:** fever of unknown origin, hemophagocytic lymphohistiocytosis, hlh-2004 protocol, hyperinflammatory state, immune system dysregulation, pancytopenia, secondary hemophagocytic lymphohistiocytosis (hlh)

## Abstract

Secondary hemophagocytic lymphohistiocytosis (HLH) is a rare, immunologically driven disorder with a high mortality rate. It is typically diagnosed on the basis of clinical and laboratory criteria. We present the case of a 53-year-old woman with multiple previous hospitalizations for fever of unknown origin and fatigue. Her workup ultimately led to a diagnosis of HLH. We discuss the presentation, diagnostic criteria, and clinical treatment of secondary HLH to guide workup and management for future patients affected by this rare disease.

## Introduction

Hemophagocytic lymphohistiocytosis (HLH) is a potentially fatal condition linked to an excessively active immune system. The pathophysiology of both the primary (familial) and secondary (acquired) forms of HLH is commonly attributed to immunologic dysregulation, which can result in overactivity of the cell-mediated immune system [1]. The resulting hyperinflammatory state can lead to cytopenias and ultimately to severe infection, sepsis, end-organ damage, and not infrequently death. 

The primary form of HLH largely affects children and is associated with several genetic mutations [2]. The secondary form of HLH has been associated with infection, autoimmunity, immune suppression, organ transplant, and malignancy; while the disease process of this potentially fatal illness is not completely understood, its pathophysiology is thought to be multifactorial [1-3]. Moreover, HLH in adults previously has been estimated to account for less than 1% of admissions to tertiary care centers, though the scarcity of data makes incidence difficult to assess accurately [3]. Diagnostic challenges of HLH derive largely from its widely variable and non-specific presentation. Delays in diagnosis and treatment may further exacerbate the effects of HLH, resulting in deterioration of clinical status. This medical instability may make certain treatment options, such as chemotherapy and stem cell transplant, more difficult to pursue. 

The most widely recognized set of diagnostic criteria for both primary and secondary HLH is based upon the HLH-2004 clinical trial [4]. These criteria were recently updated in 2024. Based on the 2024 criteria, a diagnosis of HLH is made through a molecular diagnosis consistent with HLH or by meeting five out of seven established criteria [5]. The HLH-2024 criteria remain the preferred diagnostic guidelines today. Additionally, an adult individual's risk of having HLH can be estimated using the HScore, which consists of a set of weighted clinical and laboratory criteria such as temperature, number of cytopenias, and evidence of hemophagocytosis features on bone marrow aspirate. This tool is recognized to be highly sensitive but less specific. 

Treatment for secondary HLH can include chemotherapy (such as dexamethasone and etoposide) as well as immune therapy or even hematopoietic stem cell transplant [2]. While treatment regimens and survival rate have improved, the prognosis for secondary HLH remains poor, with the mortality rate reaching up to 80% [6]. 

This report describes the case of a 53-year-old woman with multiple previous hospitalizations for fever of unknown origin and fatigue. She was eventually diagnosed with secondary HLH and treated for the condition. However, she later re-presented with recurrent HLH, and her health rapidly declined thereafter. This case demonstrates the importance of prompt workup and treatment in patients suspected of suffering from HLH. 

This case report was previously presented as a poster at Medicine Research Day in New Orleans in April 2025 and at Greater New Orleans American Physician Science Association Day in New Orleans in April 2025.

## Case presentation

A 53-year-old woman with a past medical history of hypertension and heart failure with recovered ejection fraction presented to the emergency department with weakness, fatigue, fever, chills, nausea, vomiting, and poor appetite for two months. She had been admitted to other hospitals in the months prior for similar presentations. Previous workups before this presentation had revealed severe anemia with a hemoglobin of 5.9 g/dL (59 g/L). Due to her lack of diagnosis and concern for an immunological cause of her illness, she had already completed a high-dose dexamethasone taper. Upon presentation, her vital signs were notable for fever, tachycardia, and hypotension, and she had an abnormally high lactate (Table 1).

**Table 1:** Patient vital signs and lactate upon presentation to the emergency department. MAP: mean arterial pressure

Parameters	Patient Values	Reference Range
Temperature (°C)	38.9	36.5-37.3
Heart rate (beats per minute)	132	60-100
Respiratory rate (breaths per minute)	20	12-18
Blood pressure (mmHg)	76/47	90-120/60-80
MAP (mmHg)	57	70-100
Oxygen saturation (%)	100 (on room air)	95-100
Lactate (mmol/L)	3.6	0.3-2.0

On physical exam, the patient was ill-appearing with purpura and pitting edema of her bilateral lower extremities up to the knees, as well as scattered ecchymoses on all extremities. Due to concern for pancytopenia, sepsis, and heart failure, she was admitted to the medical intensive care unit; she was also started on broad-spectrum antibiotics and pressors. Infectious diseases, gastroenterology, rheumatology, endocrinology, and hematology/oncology services were consulted. 

**Figure 1:**
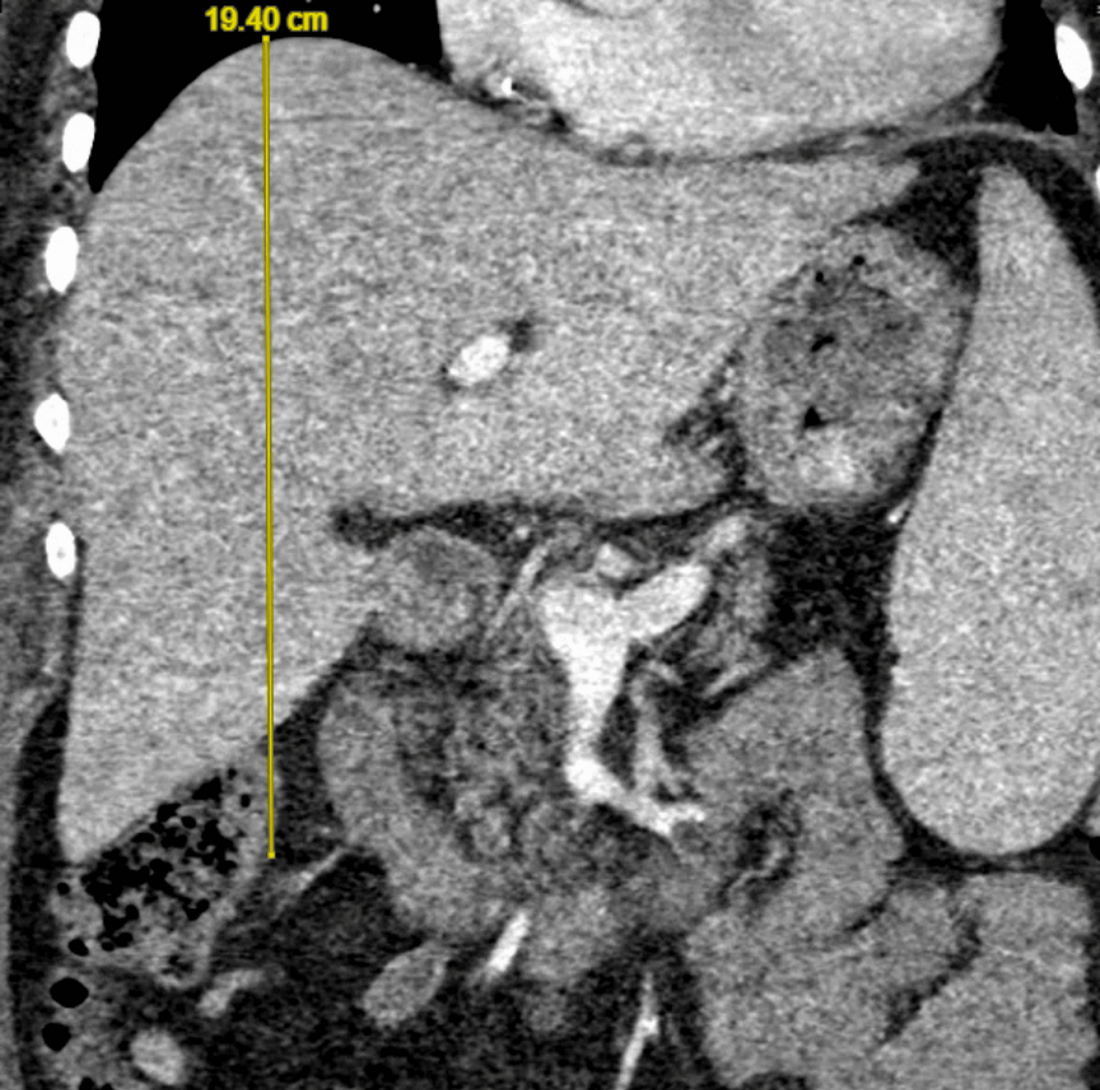
CT scan of the abdomen demonstrating hepatomegaly.

**Figure 2:**
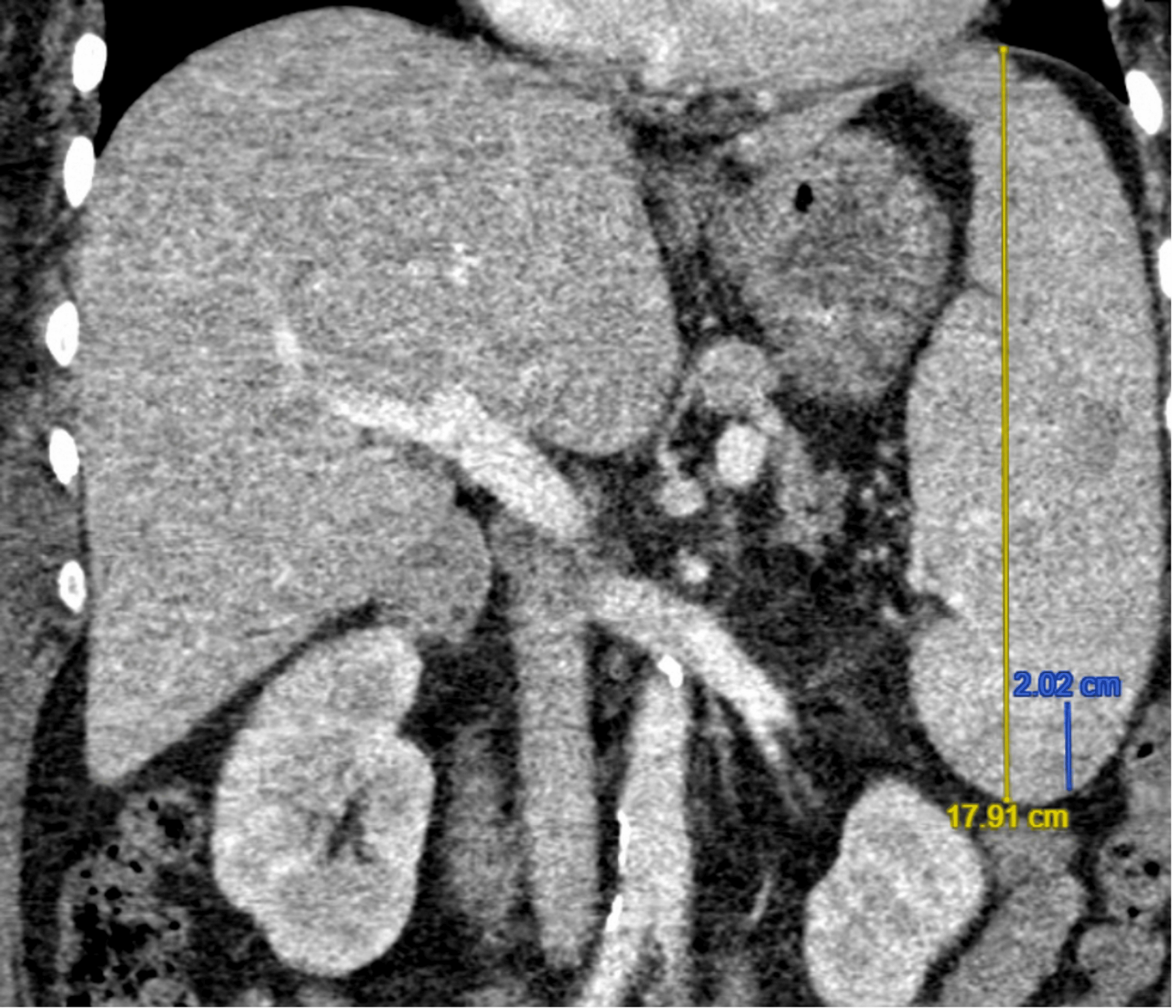
CT scan of the abdomen demonstrating splenomegaly, meeting one of the seven HLH-2024 criteria. HLH: hemophagocytic lymphohistiocytosis

Extensive infectious workup (including but not limited to chest X-ray, transthoracic echocardiogram, blood cultures, urine cultures, respiratory viral panel, and cytomegalovirus and human immunodeficiency virus testing) revealed no source of infection, and antibiotics were subsequently discontinued. Over the following days, her condition worsened. The patient underwent a comprehensive workup to evaluate for HLH. Computed tomography (CT) scan of the abdomen revealed hepatosplenomegaly; her liver measured 19.4 cm (Figure 1), and her spleen measured 17.9 cm (Figure 2). A right iliac crest bone marrow biopsy was without definitive evidence of hemophagocytosis. Flow cytometry was performed without evidence of a B- or T-cell lymphoproliferative disorder or acute leukemia. 

Several other tests were ordered as part of the patient's medical assessment. The patient met HLH-2024 diagnostic criteria (Table 2) and also had an 88-93% probability of having HLH according to the HScore (Table 3). Given that her overall clinical and laboratory workup was suspicious for HLH, her hematology/oncology team made the decision to start etoposide and dexamethasone on day six of admission. Her regimen was based on the HLH-94 protocol [4]. She received IV hydrocortisone (50 mg every six hours) and was transitioned to oral prednisone (60 mg daily). She subsequently was switched to IV dexamethasone (20 mg daily) and then to oral dexamethasone (20 mg daily). She also received three IV etoposide treatments (150 mg/m^2^) in accordance with the recommendation of etoposide treatments twice weekly for two weeks and then weekly.

**Table 2:** Clinical and laboratory workup at current presentation compared to HLH-2024 criteria. Five of seven HLH-2024 criteria [5] must be met to establish a diagnosis of HLH, including at least two cytopenias (the fourth criterion) as well as either hypertriglyceridemia or hypofibrinogenemia (the fifth criterion). The patient fulfilled six criteria, including those for fever, splenomegaly, cytopenia, hypertriglyceridemia, elevated ferritin, and elevated interleukin-2 receptor. While the patient did not meet criteria for hypofibrinogenemia, criterion 5 was still met due to her hypertriglyceridemia. HLH: hemophagocytic lymphohistiocytosis

Parameters	Patient Values	Reference Range	HLH-2024 Criteria (≥5 of 7 for diagnosis)
1. Temperature (°C)	38.9	36.5-37.3	≥38.5
2. Extension of spleen below the costal margin (cm)	≥2	<2	≥2
3. Hemophagocytosis in spleen/bone marrow/lymph node	absent	absent	present
4a. Hemoglobin (g/L)	89 (8.9 g/dL)	120-160 (12.0-16.0 g/dL)	<90 (<9.0 g/dL)
4b. Platelets (x 10^9^ /L)	27	130-400	<100
4c. Neutrophils (x 10^9^ /L)	3.1	1.8-8.0	< 1.0
5a. Fasting triglycerides (mmol/L)	6.1	<1.7	≥3
5b. Fibrinogen (g/L)	6.7	2-6	≤1.5
6. Ferritin (μg/L)	4,331	20-210	≥500
7. Soluble interleukin-2 receptor (U/mL)	≥4000	175-850	≥2400

**Table 3:** Patient’s HScore. The HScore, based on a set of clinical and laboratory criteria, can be used to estimate the probability of having HLH. The patient’s score on admission was 204 points, corresponding to 88-93% probability of having HLH.

Parameters	Patient Value	HScore
Known underlying immunosuppression	No (0 points)	No (0 points); Yes (18 points)
Temperature, °F (°C)	102.0 (38.9) (33 points)	<101.1 (<38.4) (0 points); 101.1–102.9 (38.4-39.4) (33 points); >102.9 (>39.4) (49 points)
Organomegaly	Hepatomegaly and splenomegaly (38 points)	No (0 points); Hepatomegaly or splenomegaly (23 points); Hepatomegaly and splenomegaly (38 points)
Number of cytopenias; defined as hemoglobin ≤9.2 g/dL (≤5.71 mmol/L) and/or WBC ≤5,000/mm³ and/or platelets ≤110,000/mm³	Hemoglobin 8.9 g/dL; WBC 3,100/mm³; platelets 27,000/mm³ (34 points)	1 lineage (0 points); 2 lineages (24 points); 3 lineages (34 points)
Ferritin, ng/mL (or μg/L)	4,331 (35 points)	< 2,000 (0 points); 2,000-6,000 (35 points); >6,000 (50 points)
Triglycerides, mg/dL (mmol/L)	6.1 mmol/L* *(64 points)	<132.7 (<1.5) (0 points); 132.7-354 (1.5–4) (44 points); >354 (>4) (64 points)
Fibrinogen, mg/dL (g/L)	6.7 g/L* *(0 points)	>250 (>2.5) (0 points); ≤250 (≤2.5) (30 points)
AST, U/L	19 U/L* *(0 points)	<30 (0 points); ≥30 (19 points)
Hemophagocytosis features on bone marrow aspirate	No (0 points)	No (0 points); Yes (35 points)
	204 points = 88-93% probability of HLH	Optimal cutoff is 169 points

The patient clinically improved (Table 4), and she was discharged on day 14 with an oral steroid taper (oral dexamethasone 21 mg for another six days, followed by 9 mg for 14 days and then 6 mg for 14 days) and outpatient etoposide infusions (150 mg/m^2^ once weekly for another six weeks) in accordance with HLH-directed therapy. Despite an extensive diagnostic workup, no underlying etiology, including malignancy or viral infection, could be determined.

**Table 4:** Treatment response during current admission. The patient’s response to treatment following HLH diagnosis was measured through both clinical appearance and laboratory values. She appeared clinically well and was in good spirits at discharge. She also had been consistently afebrile. Additionally, her pancytopenia improved over the course of her hospitalization, with all three cell line counts rising and stabilizing. It was not unexpected that her white blood cell count remained low, as she began chemotherapy with etoposide treatments during this admission.

Parameters	Admission	Discharge
Fever curve (°C)	38.9	36.6
Hemoglobin (g/L)	89 (8.9 g/dL)	99 (9.9 g/dL)
Platelets (x 10^9^/L)	27	72
WBC (x 10^9^/L)	3.1	3.8

Unfortunately, the patient was readmitted multiple times in the subsequent months due to neutropenic fever, shock, and sepsis (Table 5). A week after discharge from her current admission, she presented to the hospital in shock. Her workup revealed extended-spectrum beta-lactamase (ESBL) bacteremia, an acute kidney injury (AKI), and invasive aspergillosis. Her presentation was highly suspicious for recurrent HLH. Her etoposide treatments were thus discontinued early due to these life-threatening side effects. She was treated with antibiotics, including vancomycin and meropenem, as well as voriconazole. She was discharged on voriconazole for 11 weeks and an oral dexamethasone taper (5 mg daily for 10 days followed by 2.5 mg daily for seven days). 

**Table 5:** Clinical course and treatments across multiple admissions. The patient’s clinical course is summarized across multiple admissions, including both the admission during which she was officially diagnosed with HLH (first/current admission) as well as prior admissions and subsequent admissions. During her third and final admission, her presentation was consistent with HLH relapse. Despite treatment, her condition continued to worsen. After a goals of care discussion, the patient decided to discontinue life-prolonging care in favor of focusing on comfort measures, and she was discharged home with her family on hospice.

Timeline	Events
Prior admission (Day 0)	Patient admitted for generalized weakness, abdominal pain, fever, and pancytopenia requiring multiple transfusions. Bone marrow biopsy unremarkable. Started on IV dexamethasone and transitioned to oral.
Day 19	Patient discharged with dexamethasone taper (starting at 10mg daily) and hematology/oncology follow-up due to concern for underlying hematologic or oncologic process.
First (current) admission (Day 46)	Patient admitted for pancytopenia and shock with abdominal pain. Negative infectious and malignant workup. Comprehensive HLH workup resulted in HLH diagnosis. Patient started high-dose steroid therapy and etoposide infusions with subsequent clinical and laboratory improvement.
Day 60	Patient discharged with dexamethasone taper (starting at 21mg daily) and outpatient etoposide infusions.
Second admission (Day 67)	Patient admitted for fever, hypotension, and sepsis. Workup revealed ESBL bacteremia, cellulitis, and invasive aspergillosis treated with antibiotics (vancomycin and meropenem) and anti-fungals (voriconazole). Also found to have AKI likely due to hypoperfusion injury. Etoposide treatments stopped. Continued on steroids.
Day 86	Patient discharged to long-term acute care facility (LTAC) in stable condition with continued dexamethasone taper (starting at 5mg daily) and outpatient voriconazole treatment.
Third admission (Day 203)	Patient admitted for abdominal pain, pancytopenia, shock, and sepsis requiring pressor support and ICU stay. Presentation consistent with HLH relapse. Patient started on anakinra and dexamethasone. Anakinra was stopped due to little response. Patient’s clinical condition continued to worsen. Goals of care discussion with patient and family. Decision made to withdraw life-prolonging treatment and to focus on comfort care.
Day 238	Patient discharged home with family on hospice.

The patient’s next admission occurred nearly four months later. She presented with abdominal pain, pancytopenia, and shock similar to her previous HLH flare. She temporarily required pressors and an ICU stay but was eventually stabilized. 

Given that she had previously developed significant and life-threatening toxicities leading to early termination of her etoposide medication regimen, pursuing etoposide was not a viable option. Thus, an IL-1 receptor antagonist, anakinra (10 mg/kg per day), was started as a temporizing agent. Unfortunately, she showed little response, as she continued to have fevers, fluctuating hypotension, pancytopenia, and rising ferritin levels. Her anakinra treatments were stopped after less than a week. She was also started on hydrocortisone (50 mg every six hours) and then transitioned to dexamethasone (10 mg every 12 hours). 

Due to her worsening clinical condition, the patient wished to discontinue further therapy in favor of concentrating on comfort care. With the help of her palliative care team, she was discharged with plans for home hospice less than eight months after her initial diagnosis. 

## Discussion

The exact etiology of secondary HLH is still not fully understood. Indeed, its clinical presentation can be non-specific and perplexing, leading to possible delays in diagnosis and treatment. As previously noted, early diagnosis can influence patient outcome [7], thus making reliable diagnostic criteria even more important. 

While the HLH-2004 criteria have historically been the most widely accepted diagnostic tool for HLH, their use in diagnosing secondary HLH in particular has become somewhat controversial. This controversy stems from their origins as the inclusion criteria for a pediatric clinical trial that mostly examined patients afflicted by primary HLH [8] rather than secondary HLH, which is the more common form in adults. 

As noted previously, the HLH-2004 criteria were recently updated to the HLH-2024 criteria, which now exclude natural killer (NK)-cell activity from the recommended laboratory measurements. Given the differences in pathogenesis between primary and secondary HLH [3], it would be beneficial to investigate the development of a revised set of guidelines and validated diagnostic criteria specific to secondary HLH. Some work has already been done on this front, including the development of a diagnostic score known as the HScore [9,10]. Currently, the HLH-2024 criteria are still generally preferred. 

This patient met six out of seven HLH-2024 criteria, including those for fever, splenomegaly, cytopenias of two cell lines, hypertriglyceridemia, elevated ferritin, and elevated interleukin-2 receptor. The only HLH-2024 criterion that this patient did not fulfill was evidence of hemophagocytosis. However, despite the fact that she met diagnostic criteria, her case was a perplexing one, likely owing in part to the rarity of this illness as well as its non-specific presentation. Greater awareness of secondary HLH and its presentation may be beneficial in mitigating delayed recognition of the disease. 

Furthermore, this patient’s recurrence complicated an already challenging case. She was no longer a candidate for etoposide, one of the mainstays of HLH treatment, and showed a minimal response to other attempted therapies. A firmer understanding of the underlying etiology in this case could have led to opportunities for more targeted therapy. Further work needs to be done to understand the varied etiology underlying secondary HLH. 

Finally, modified treatment algorithms specific to secondary HLH in adults would be worth investigating. Current treatment approaches are largely adapted from pediatric guidelines; proposed adjustments for treatment of HLH in adults include dose reductions and individualized tailoring [11]. Treatment modifications should also be considered in patients for whom typical treatments are incompatible or those who show signs of relapsed disease.

Few case reports on recurrent adult HLH exist. A report by Machaczka et al. details the successful treatment of recurrent malignancy-associated HLH in a young woman, which included a stem cell transplant and donor lymphocyte infusion [12]. However, in this case, the patient’s HLH was known to be associated with malignancy, whereas for our patient, the etiology was unknown. 

Overall, relapsed and refractory HLH has poor outcomes in the adult population. Hematopoietic stem cell transplant (HSCT) is a mainstay of treatment in these cases, regardless of the underlying cause of HLH, and may result in response rates up to nearly 50% [13]. However, stabilizing and medically optimizing patients prior to HSCT remains challenging. New drugs such as emapalumab and ruxolitinib are emerging as potential bridges to HSCT, with partial responses ranging from 14.2% to 100% [11,13,14]. Both of these medications were considered for our patient prior to her decision to pursue comfort care. 

Other emerging therapies include alemtuzumab, tocilizumab, and anakinra, which have shown promising results [11,13,14]. One study showed that anakinra, a medication that is generally tolerated well, was effective in 90.5% of patients, with patients showing fever resolution within a median of one day as well as decreased ferritin and C-reactive protein levels [15]. Anakinra was trialed in our patient during her last admission, but unfortunately, she showed little response to the drug, resulting in discontinuation. 

Overall, HLH continues to present a significant challenge for patients and providers from both a diagnostic and treatment perspective. Despite its often non-specific presentation, providers should maintain a high index of suspicion for HLH in patients with recurrent fevers of unknown origin accompanied by cytopenias. Thereafter, providers should quickly begin an HLH workup as well as look into potential causes and individualized treatments. 

## Conclusions

HLH is a complex and potentially deadly proinflammatory disorder. Our patient fulfilled six of the seven HLH-2024 diagnostic criteria. The exact cause of HLH in this patient remains elusive and likely never will be fully elucidated. Although a diagnosis was reached and a management plan initiated, her condition continued to lead to extended hospital admissions and an unfavorable outcome. Due to the life-threatening nature of this disease, timely diagnosis is imperative to improving patient outcomes. Clinicians should consider HLH as a potential diagnosis in patients with recurrent fevers and cytopenias, prompting hematological workup. Additionally, clinicians who suspect HLH should investigate possible underlying etiologies, such as malignancy or infection.

This patient suffered multiple adverse effects not only from her illness but also from her treatment, and eventually she chose to pursue comfort care rather than further therapy. Attaining overall more favorable prognoses will require continued research into this disease, including the development of both validated diagnostic criteria for secondary HLH as well as improved guidelines for therapy regimens.
